# Estrogen receptor alpha deletion enhances the metastatic phenotype of Ron overexpressing mammary tumors in mice

**DOI:** 10.1186/1476-4598-11-2

**Published:** 2012-01-06

**Authors:** Aaron M Marshall, Rebecca J McClaine, Devikala Gurusamy, Jerilyn K Gray, Kara E Lewnard, Sohaib A Khan, Susan E Waltz

**Affiliations:** 1 Department of Cancer and Cell Biology, University of Cincinnati, College of Medicine, Cincinnati, OH 45267-0521, USA; 2 Department of Surgery, University of Cincinnati, College of Medicine, Cincinnati, OH 45267-0521, USA; 3 Research Service, Cincinnati Veterans Affairs Medical Center, Cincinnati, OH, USA

**Keywords:** Ron Receptor, MST1R, Hepatocyte growth factor-like protein, breast cancer, estrogen receptor

## Abstract

**Background:**

The receptor tyrosine kinase family includes many transmembrane proteins with diverse physiological and pathophysiological functions. The involvement of tyrosine kinase signaling in promoting a more aggressive tumor phenotype within the context of chemotherapeutic evasion is gaining recognition. The Ron receptor is a tyrosine kinase receptor that has been implicated in the progression of breast cancer and evasion of tamoxifen therapy.

**Results:**

Here, we report that Ron expression is correlated with *in situ*, estrogen receptor alpha (ERα)-positive tumors, and is higher in breast tumors following neoadjuvant tamoxifen therapy. We also demonstrate that the majority of mammary tumors isolated from transgenic mice with mammary specific-Ron overexpression (MMTV-Ron mice), exhibit appreciable ER expression. Moreover, genetic-ablation of ERα, in the context of Ron overexpression, leads to delayed mammary tumor initiation and growth, but also results in an increased metastasis.

**Conclusions:**

Ron receptor overexpression is associated with ERα-positive human and murine breast tumors. In addition, loss of ERα on a Ron overexpressing background in mice leads to the development of breast tumors which grow slower but which exhibit more metastasis and suggests that targeting of ERα, as in the case of tamoxifen therapy, may reduce the growth of Ron overexpressing breast cancers but may cause these tumors to be more metastatic.

## Background

To date, the most successful pharmacological therapies specifically targeting breast cancer include anti-estrogens and receptor tyrosine kinase (RTK) modulating drugs [1]. Accordingly, there have been numerous studies examining signaling paradigms between estrogen and RTK signaling pathways [2-4] which have provided evidence that RTKs are able to activate estrogen receptor alpha (ERα) in breast cancers independent of its ligand estrogen. This activation of ERα by RTKs leads to an ERα transcriptional program that enhances cell survival. The dependency of this activation on the RTK ligand is still an area of active investigation. More importantly, however, this signaling crosstalk between RTKs and ERα may predict resistance to anti-estrogen hormonal therapies, including tamoxifen [2,5]. Specifically, studies have shown that activation of EGFR, Her2, cMet, IGFR, RET and recently, Ron RTK, lead to phosphorylation and activation of ERα which enhances survival of breast cancer in the presence of anti-estrogen therapy [4,6-8].

Ron is a cell surface RTK related to the c-Met receptor that has been identified as an oncogene in the development and growth of human epithelial tumors [9]. In the developing mammary gland, Ron is expressed during the pubertal growth stages, and then again during pregnancy and lactation, and its expression remains low in quiescent glands [10]. In normal mammary development, genetic loss of Ron signaling has been shown to alter mammary gland branching morphogenesis during puberty [10]. In cell lines, wild-type Ron overexpression is associated with induction of oncogenic properties, including malignant transformation, proliferation, and migration [11]. Overexpression of Ron in transgenic mouse models of both lung and breast cancer is associated with tumorigenesis in both organs [12,13]; while deletion of Ron in transgenic mice expressing polyoma virus middle T antigen caused a significant reduction in breast tumor formation and growth [14]. Additionally, Ron is known to be upregulated in a number of human epithelial cancers, including breast, lung, stomach, colon, pancreas, and prostate. Specifically, Ron is highly expressed in approximately 50% of human breast cancers [15]. Given the important role of Ron in human and mouse tumorigenesis, identifying the functions and signaling pathways associated with this RTK may provide essential clues to combat disease progression.

Recent data has shown that Ron activation leads to the phosphorylation and activation ERα. In this case, exogenous overexpression of Ron or ligand activation of endogenous Ron led to enhanced survival of several ERα-positive breast cancer cell lines in the presence of the anti-estrogen therapy tamoxifen [4]. Estrogen receptor alpha and its ligand estrogen are important regulators of mammary gland development and breast carcinogenesis. During development ERα is critical for peripubertal ductal elongation, and is a permissive factor in alveolar expansion during pregnancy [16-19]. In cancer, ERα transcriptional programming provides survival and growth stimulation that is advantageous for tumors and the majority of human breast cancers express ERα. Consequently, ERα is the target of a family of anti-estrogen pharmacological compounds including tamoxifen. Although tamoxifen treatment is often initially successful at preventing the proliferative effect of estrogen on ERα, the preponderance of patients develop tamoxifen-resistance [5] and the recurrent tumors tend to be more aggressive [17]. Tamoxifen resistance has become a major obstacle to effective treatment of ERα-positive breast cancer.

Complementing previously published work, we show here that Ron is overexpressed and correlated with early stage ERα-positive breast cancers. To directly demonstrate the effect of ERα inactivation has on breast cancer, in the presence of Ron, we have created ERα -replete and ERα -conditionally deficient animals on a Ron overexpressing background. We show for the first time that mammary-specific ERα deletion increases breast tumor latency, but also leads to a more metastatic phenotype. Interestingly, these studies suggest a negative consequence of anti-estrogen therapy in Ron expressing tumors on the promotion of metastasis.

## Results

### Correlations between MST1R/Ron, ER & Breast Cancer Staging

**Figure 1:**
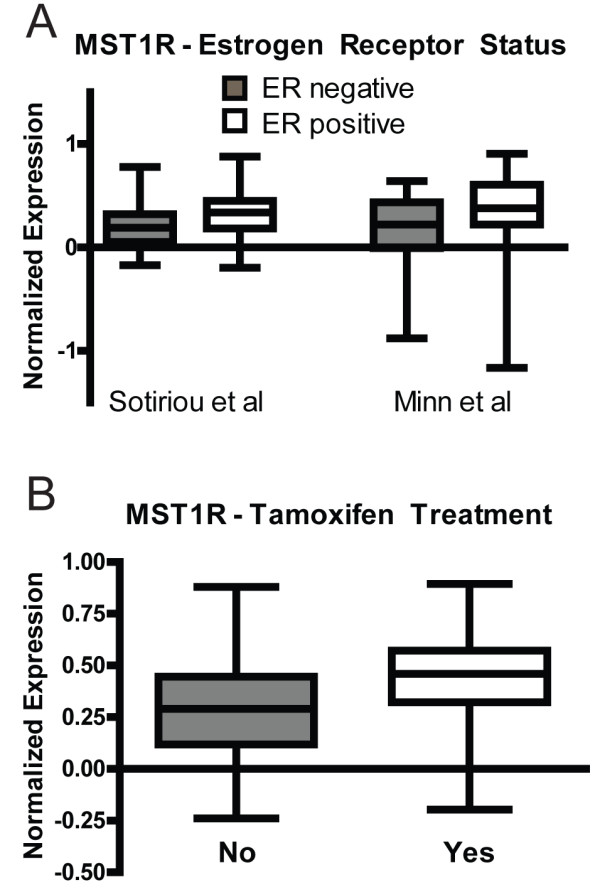
**MST1R (Ron) mRNA expression in publically available data sets**. (**A**) Comparison of MST1R expression in human breast tumor tissue between estrogen receptor positive and estrogen receptor negative tumors. Shown are box and whisker plots from two independent data sets; line represents the median, box represents interquartile range and whiskers represent min/max (p < 0.01 for each comparison utilizing the Mann-Whitney test). (**B**) Utilizing the Sotiriou *et al. *data set, we show MST1R expression in breast cancer tissue between patients who received neoadjuvant tamoxifen (Yes) versus those who did not receive neoadjuvant therapy (No) (p < 0.01, Mann-Whitney test).

Previous studies have shown that Ron activation leads to phosphorylation and activation of ER [4]. However, no studies have shown an association between Ron and ER in the human patient population. To accomplish this we first mined publically available data in Oncomine. Two independent microarray studies demonstrated that Ron (MST1R) expression positively correlates with ER-positive breast cancers (Figure 1A) [20,21]. Furthermore, Ron expression is also increased in patients following neoadjuvant tamoxifen therapy (Figure 1B).

**Figure 2:**
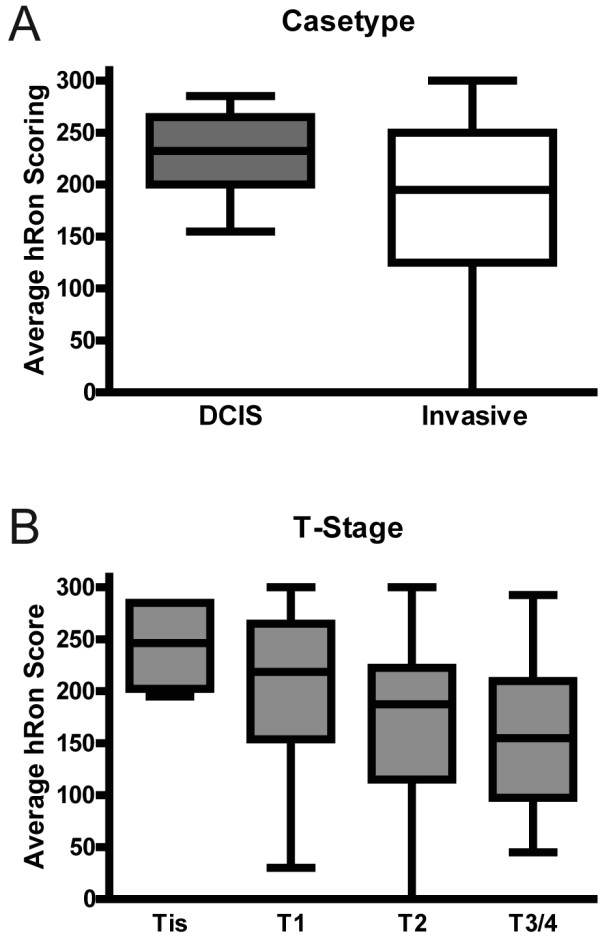
**Tissue array immunohistochemical staining for Ron**. (**A**) Comparison of Ron staining score in DCIS versus invasive cancer subtypes (p < 0.01, Mann-Whitney test, n = 250). (**B**) Comparison of Ron staining score among T-stage (Tis = carcinoma *in situ*, T1 = tumor < 20 mm, T2 = tumor > 20 mm and < 50 mm and T3/4 = tumor > 50 mm). Tis versus T1, and T2 versus T3/4 did not reach statistical significance, but all other pairwise comparisons had p-values < 0.05 by Kruskal-Wallis test followed by Dunn's multiple comparisons (n = 250).

Ron receptor overexpression has been reported in approximately half of breast cancers [15,22]. However, little information is known regarding the association of Ron overexpression with a particular subtype or stage of human cancer. To address this, immunohistochemistry for Ron was performed on three independent breast cancer tissue arrays. The arrays contained 250 human breast tumor specimens. Ron staining and expression were determined as previously described [22,23]. As depicted in Figure 2A, Ron was found to be more highly expressed in carcinomas *in situ *versus invasive carcinomas. In an analysis of Ron expression by T-stage, Figure 2B demonstrates that Ron is inversely associated with T-stage with Ron expression being highest in the *in situ *tumors (Tis), especially when compared to T2 and T3/4. T3 and T4 were grouped together due to the small number of T4 tumors present on the array. Tis versus T1, and T2 versus T3/4 did not reach statistical significance, but all other pairwise comparisons had p-values < 0.05. Altogether, the data suggests Ron is prominently featured in early stage ER+ tumors.

### Tumor Characterization in WAP-Cre/ER^fl/fl^/MMTV-Ron Mice

**Figure 3:**
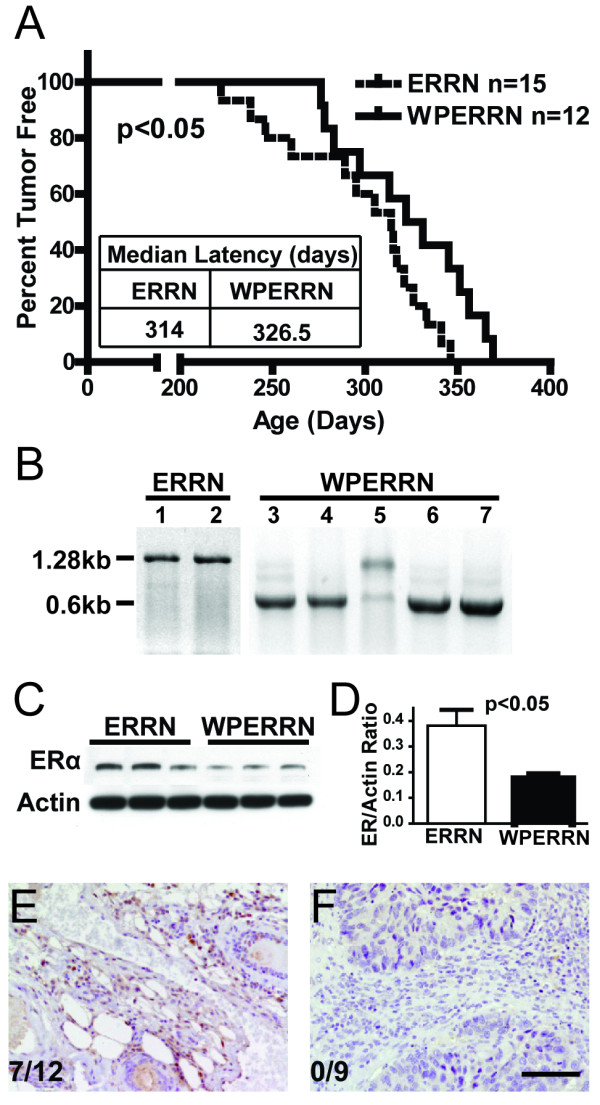
**Tumor characteristics of ERRN and WPERRN mice**. (**A**) Tumor latency plotted on a Kaplan-Meier curve showing percentage of ERRN (n = 15) versus WPERRN (n = 12) mice that were tumor free versus time (p < 0.05, log-rank test). (**B**) DNA bands resulting from conventional PCR on genomic DNA isolated from tumor tissue of ERRN and WPERRN mice. Cre-mediated recombination results in ERα knockout tumor tissue, shown by the presence of a 0.6 kb PCR product; while the wild-type ERα product is 1.28 kb. (**C**) Western blot of ERα on tumor tissue lysates from ERRN and WPERRN mice with (**D**) quantification. (**E, F**) Immunohistochemical staining for ERα on mammary tumor sections from ERRN and WPERRN mice. Numbers represent the proportion of animals in each genotype that developed ER-positive tumors. Scale bar = 50 μm.

Since Ron is associated with early stage ER+ tumors, a set of genetic experiments was performed to test the hypothesis that tumor progression is altered in mice where ERα is non-functional and Ron is overexpressed. To accomplish this, the MMTV-Ron driven mouse model of breast cancer was utilized. As previously reported, MMTV-Ron mice develop breast cancer with 100% penetrance [13]. To generate ER^fl/fl^/MMTV-Ron mice (ERRN), MMTV-Ron mice were crossed to ER^fl/fl ^mice. To create mice on the MMTV-Ron background that are deficient in ERα protein, we then crossed ERRN mice with transgenic mice expressing Cre recombinase under the direction of the whey acidic protein promoter (WAP-Cre) to obtain WAP-Cre Mice/ER^fl/fl^/MMTV-Ron (WPERRN) mice and littermate controls (ERRN). The ERRN control mice develop mammary tumors with 100% penetrance and a median time to palpable tumor of 314 days (Figure 3A). The WAP-Cre Mice/ER^fl/fl^/MMTV-Ron (WPERRN) mice also develop mammary tumors with 100% penetrance; however the WPERRN mice exhibited a significant increase in tumor latency (p < 0.05, log-rank test) compared to controls (Figure 3A). The median tumor latency in this group is increased by 12.5 days to 326.5 days compared to mammary tumors from ERRN mice.

To verify Cre-mediated deletion of a 680 bp region of the ERα allele, conventional PCR was performed on DNA isolated from excised mammary tumors of ERRN and WPERRN mice. All ERRN mice had the endogenous full length 1280 bp wild-type ERα allele, represented by lanes 1 and 2 in Figure 3B. Of the 16 mammary tumors analyzed from WPERRN mice, all had both the wild-type ERα allele (1280 bp band) and the knockout ERα allele (600 bp band). Four animals had a wild-type band that was greater than 2-fold stronger than the knockout band as shown in lane 5 (Figure 3B) suggesting limited ERα deletion in this tumor. These mice were excluded from all analyses, including Figure 3A. The remaining 12 tumors had a greater than 5-fold more intense knockout band compared to the wild-type band and are represented by lanes 3-4, 6-7 (Figure 3B). To determine the extent of ERα protein depletion, whole tissue lysates from ERRN and WPERRN mammary tumors were examined by Western analyses. ERα protein was reduced approximately 50% as shown in Figure 3C and quantified in Figure 3D. Given the heterogeneous origin of the mammary tumor tissue, and the expression pattern of whey acidic protein, it is not surprising that ERα is still present, albeit at lower levels. To verify that ERα was indeed deleted from the tumors of WPERRN mice, and to document the percentage of ERRN mice that developed ERα-positive tumors, we performed immunohistochemistry on tumor sections. Using a scoring system previously described for determining ER-positivity [24], we determined that in ERRN mice, ERα-positive tumors occur approximately 58% of the time (7/12) (Figure 3E). In contrast, none of the nine WPERRN mice had ERα-positive tumors (Figure 3F).

### Analysis of proliferation and death rates in WPERRN and ERRN tumors

**Figure 4:**
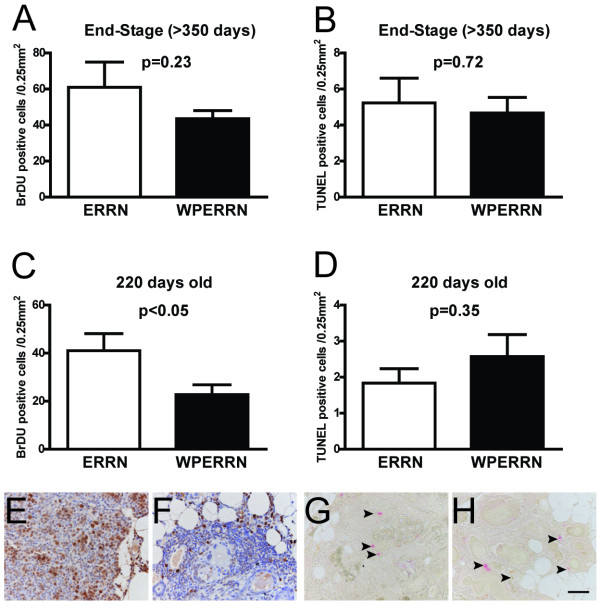
**Analysis of proliferation and cell death in ERRN and WPERRN tumors**. BrDU immunohistochemical staining was performed on end-stage tumors (> 2 cm^3^) (**A**) and mice with neoplasias at 220 days old (**C**). Bars represent the average number of positive cells per field of view (0.25 mm^2^). Pictures (**E**) and (**F**) correspond to ERRN (n = 7) and WPERRN (n = 7) representative examples, respectively. TUNEL staining was performed on end-stage tumors (**B**) and 220 day old mice (**D**). Bars represent the average number of positive cells per field of view (0.25 mm^2^). Pictures (**G**) and (**H**) correspond to ERRN (n = 7) and WPERRN (n = 7) representative examples, respectively. Scale bar = 50 μm.

The increase in latency of WPERRN tumors compared with ERRN tumors suggests that tumor cell proliferation and/or apoptosis may be altered. To examine this, we performed BrDU and TUNEL staining on end-stage mammary tumors, which was defined as a primary tumor reaching ~2.5 cm^3 ^in size. Interestingly, BrDU incorporation was not significantly different in WPERRN mice versus ERRN mice (Figure 4A), nor was TUNEL staining (Figure 4B). To more accurately assess the proliferation and apoptosis rates during tumor initiation, we harvested mammary glands from age matched WPERRN and ERRN mice at 220 days of age and stained for BrDU and TUNEL. This time point was determined to be optimal for obtaining mammary glands with hyperplasia, but prior to palpable tumor formation. At 220 days old, WPERRN mammary epithelial cells displayed significantly lower BrDU incorporation than in ERRN mice (Figure 4C). Representative images for 220 day old BrDU staining are shown in Figure 4E and 4F. TUNEL staining at 220 days old was low in these neoplastic regions and was not different between ERRN and WPERRN animals (Figure 4D, G and 4H).

### Alterations in metastatic burden in WAP-Cre/ER^fl/fl^/MMTV-Ron Mice

**Figure 5:**
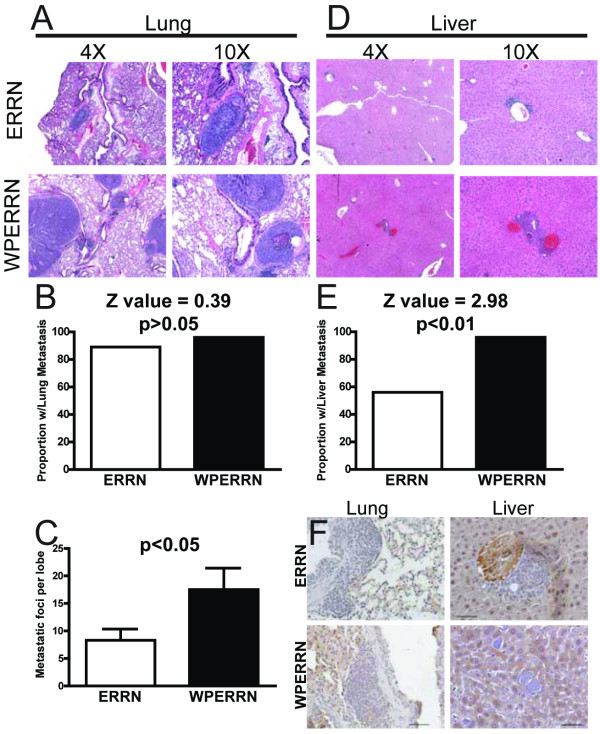
**Metastatic burden analysis on the lungs and livers of ERRN and WPERRN mice**. H&E photomicrographs of a lung (**A**) and liver (**D**) metastasis is shown for ERRN and WPERRN genotypes. Proportion of mice having either lung (**B**) or liver (**E**) metastases is shown for ERRN (white bars n = 24) and WPERRN (black bars n = 24) mice (z-test). (**C**) Bars represent the average number of metastatic foci found per lobe of a lung in ERRN and WPERRN animals (t-test). (**F**) Immunohistochemical staining for ER in the metastases of ERRN and WPERRN lung and liver.

The liver and lungs were targeted for metastatic foci examination and quantification. Previously, we reported that MMTV-Ron transgenic mice develop metastases to these two organs [13]. The lungs and livers were excised and fixed in formalin at the time of sacrifice. The lungs of ERRN and WPERRN mice both contained metastases that were histologically similar (Figure 5A). To quantify the overall metastatic burden, we first determined the metastatic rate to the lungs using a binary system (+ or -). The proportion of mice with metastases between ERRN and WPERRN mice was not statistically different (89% and 96% respectively; Z value = 0.39) (Figure 5B). However, histological examination of the number of metastatic foci in a single lobe revealed that the WPERRN mice carried a higher metastatic burden in the lungs than the ERRN mice (Figure 5C). Immunohistochemical staining for cytokeratin 18 verified the origin of the metastases as from the breast (data not shown). Next we examined the livers of ERRN and WPERRN mice. As with the lungs, both genotypes had liver metastases that were histologically indistinguishable from each other (Figure 5D). Again using a binary scoring system for the presence of liver metastases, we determined that 56% of ERRN mice and 96% of WPERRN mice had metastatic liver foci (P < 0.01 Z value = 2.98) (Figure 5E). We confirmed the origin of the foci by immunostaining for cytokeratin 5, which was determined to be present in the MMTV-Ron mammary tumors and not in hepatocytes (data not shown). Next, we sought to determine the extent of ER positivity in the metastases. The metastases in all genotypes and both tissues stained negative for ER (Figure 5F). This reduction in the number of metastases may be reflective of the proportion of ER negative tumors in the different genotypes. Overall, we demonstrate the MMTV-Ron driven tumors that have deleted ERα have a higher metastatic burden to the lungs and liver.

### ERα deletion in MMTV-Ron tumors is associated with decreased expression of ER-dependent genes

**Figure 6:**
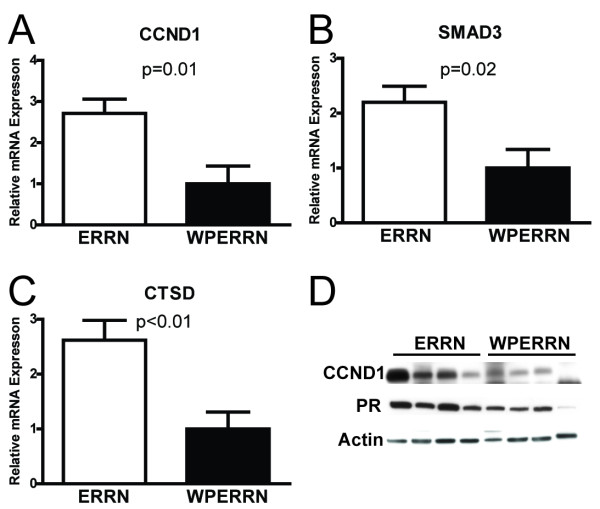
**Expression changes of ER-regulated genes**. (**A, B, C**) Bars represent the relative mRNA expression levels between ERRN (n = 7) and WPERRN (n = 7) tumor lysates, quantified by qPCR. The ER-regulated genes (**A**) CyclinD1 (CCND1) (**B**) SMAD3 and (**C**) cathepsin D (CTSD) were analyzed (t-test). (**D**) Protein lysates were also analyzed by Western blotting for expression of cyclin D1 and progesterone receptor.

To explore a potential mechanism by which ERα deletion leads to increased latency and enhanced metastasis, we evaluated the expression of ERα-dependent target genes by real-time PCR and western blotting. Cyclin D1 (CCND1), mothers against decapentaplegic homolog 3 (SMAD3) and cathepsin D (CTSD) are genes known to be stimulated by ERα activation [25-27]. Tumor tissue representing WPERRN and ERRN mice was homogenized and the total RNA isolated. Real-time PCR was performed on seven independent samples, plated in duplicate. In WPERRN mice the expression of CCND1, SMAD3 and CTSD were all decreased compared to ERRN animals by approximately 60% (P < 0.01, 0.02, 0.005 respectively) (Figure 6A, B &6C respectively). Whole protein lysates from WPERRN and ERRN tumors were also analyzed for expression of cyclinD1. Expression of cyclinD1 was decreased in WPERRN tumors, which correlates with the gene expression studies (Figure 6D). Furthermore, progesterone receptor, a well-known protein product of ER signaling was also decreased to a similar extent as cyclinD1 (Figure 6D).

### Pure ER^fl/fl^Ron and ER^-/-^Ron cells recapitulate tumor growth phenotypes

**Figure 7:**
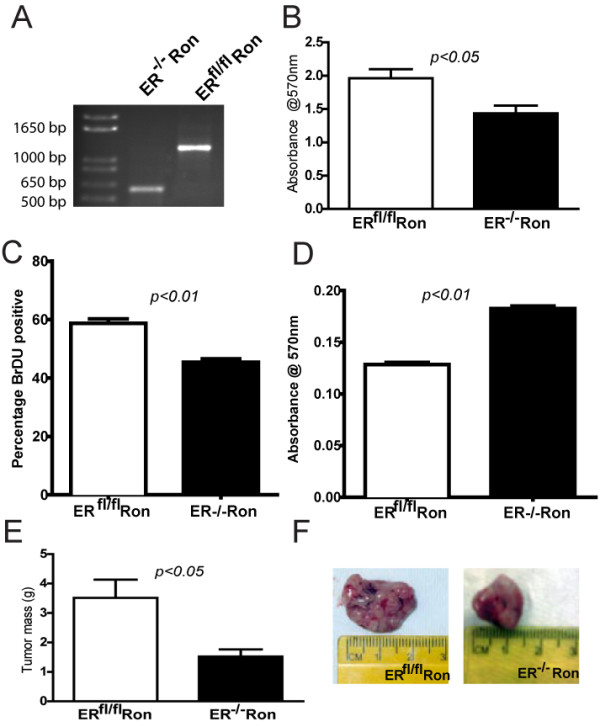
**Characterization of ER^fl/fl^Ron and ER^-/-^Ron cells**. (**A**) PCR analysis of genomic DNA confirming the presence of the wild-type ERα allele (1280 bp) in ER^fl/fl^Ron cells and the knockout allele (600 bp) in ER^-/-^Ron cells. (**B**) Bars represent the absorbance measured at 570 nm following MTT assay on ER^fl/fl^Ron and ER^-/-^Ron cells (n = 8). (**C**) Bars represent the percentage of BrDU positive cells per 10 × field of ER^fl/fl^Ron and ER^-/-^Ron cells following BrDU incorporation (n = 6 independent experiments with 6-8 fields counted per genotype/experiment). (**D**) Bars represent the absorbance measured at 570 nm following a transwell migration assay on ER^fl/fl^Ron and ER^-/-^Ron cells (n = 4 independent experiments performed in triplicate). (**E**) Bars represent the mean mass of the tumors established from orthotopic injections of ER^fl/fl^Ron and ER^-/-^Ron cells (n = 7 mice per cell line) into the fat pads of athymic nude mice a time of sacrifice (10 weeks post injection). All means were compared by student's t-test. (**F**) Representative photographs of tumors established from orthotopic injections of ER^fl/fl^Ron and ER^-/-^Ron cells into the fat pads of athymic nude mice.

Although, WPERRN animals had delayed tumor latency, the ERα deletion did not display 100% penetrance (see Figure 3B). Therefore to confirm the tumor growth phenotype, we established a cell line derived from an ER^fl/fl^MMTV-Ron tumor. We subsequently infected the ER^fl/fl^MMTV-Ron tumor cells with an adenovirus expressing Cre-recombinase and GFP or GFP alone. GFP-positive cells were isolated by FACS and cultured to establish ER^fl/fl^Ron cells and ER^-/-^Ron cells. PCR analysis confirmed the Cre-mediated deletion of a 600 bp region of exon 3 of ERα (Figure 7A). This deletion was still evident at passage 23, with no contaminating wild-type ERα allele detected (data not shown); and all subsequent experiments were performed between passages 5 and 15. First, the proliferative rates of ER^fl/fl^Ron cells and ER^-/-^Ron cells were compared by the MTT assay and BrDU incorporation. Confirming the phenotype of ERRN and WPERRN mice, the ER^fl/fl^Ron cells displayed an increased proliferative rate compared to ER^-/-^Ron cells, by both MTT (Figure 7B) and BrDU incorporation experiments (Figure 7C). No differences in cell death or apoptosis were observed between the ER^fl/fl^Ron and ER^-/-^Ron cells as judged by Annexin V and propidium iodide (PI) staining (data not shown). Next, to detect differences in metastatic potential, migration experiments were performed. Again supporting the phenotype observed in mice, the ER^-/-^Ron cells migrated more aggressively across a transwell membrane (Figure 7D). The ER^-/-^Ron cells also invaded significantly more on transwell membranes which were coated with Matrigel^®^, a basement membrane protein (data not shown). Furthermore, when these same cells were injected into the mammary fat pads of adult athymic nude mice, larger tumors were observed in ER^fl/fl^Ron cell orthotopic injections compared to ER^-/-^Ron cells (10 weeks) (Figure 7E &7F). Metastases were not found in the lungs or livers of either group of nude mice at the time of sacrifice; prohibiting analysis of metastatic burden.

## Discussion

In this study we have genetically deleted ERα in a relevant model of murine mammary cancer (MMTV-Ron). We have shown that Ron expression is associated with early stage ER+ human breast cancers with the highest levels of Ron observed in carcinomas *in situ*. Ductal carcinomas *in situ *(DCIS) represent an increasing population of new patient diagnoses. Currently, 20-30% of new cases are classified in this type [28] and evidence exists that DCIS is a precursor lesion for invasive cancer [28]. Therapeutic options for treating DCIS depend on hormone status and the patient's wishes. Patients with ER+ DCIS usually undergo lumpectomy and receive tamoxifen as a neoadjuvant or adjuvant therapy [28]. Since Ron is often overexpressed in these types of tumors, and Ron has previously been shown to mediate tamoxifen insensitivity, our data suggest that Ron may be an important biomarker for predicting chemotherapeutic resistance.

Previous work has shown that estrogen acts as a carcinogen via three independent mechanisms: (1) stimulation of proliferation through receptor signaling, (2) direct genotoxic effects via cytochrome P450, and (3) induction of aneuploidy [29]. In addition, the role of estrogen in stimulating proliferation in both normal and neoplastic tissue is well documented (30). Proliferation due to estrogen is dependent on ERα. In this study, we were able to remove estrogen-ERα mediated proliferation by genetic manipulation. During tumor development, we observed that ERα deficient mammary tumors have a lower proliferative rate as measured by BrDU incorporation. Our studies also document an increase in tumor latency in these mice. Furthermore, cells derived from these tumors and manipulated to re-create the ER-replete and ER-knockout conditions recapitulated the proliferative phenotype observed in these animals. This confirms that in our MMTV-Ron driven model of carcinogenesis, estrogen-ERα signaling plays a proliferative role that can be attenuated by ERα deletion.

Interestingly, our studies provide important information related to the loss of ERα in the context of Ron overexpression, in that while mammary tumors with this loss grew slower, the tumors were overall more metastatic. This data suggest that the context in which ERα is deleted (or inhibited as in the case of anti-estrogen therapy akin to clinical studies), Ron may play an important role in regulating tumor progression and metastasis. In this case, a suggestion of dually targeting Ron and ERα may prove advantageous. While further studies are warranted to understand the mechanisms associated with the enhanced metastatic phenotype, a potential mechanism is that in WPERRN tumors compared to controls, the levels of the ER-regulated genes SMAD3 and cathepsin D are lower. Decreased expression of SMAD3 has been shown to uncouple TGF-beta signaling, resulting in a local immunosuppressive environment [30]. Cathepsin D functions primarily as a lysosomal peptidase in the processing of antigen for presentation. Inhibition or decreased expression of cathepsin D results in decreased antigen processing and presentation [31]. In conjunction with ER-dependent gene changes, Ron activation has been shown to play an important role in the suppression of innate immunity [32,33]. Taken altogether, the decreased expression of SMAD3 and cathepsin D due to ER loss, and concomitant overexpression of Ron may provide an environment favoring immune system evasion, which may be contributing to the enhanced metastatic phenotype. Testing this hypothesis, preliminary experiments showed that F480 staining in primary tumors was not significantly different between ERRN and WPERRN genotypes (data not shown). Further investigation is needed to verify and expand upon these findings.

The increased metastatic burden found in the WPERRN mice is interesting on two levels. First, these mice had neoplastic lesions that were slower to proliferate than ERRN neoplasias. There has been recent evidence suggesting that slower growing tumors metastasize at a higher rate [34]. The second level where the alteration in metastasis is interesting is with respect to anti-estrogen endocrine therapies. Tamoxifen and other anti-estrogen therapies ultimately fail due to acquired resistance. The endocrine therapy selects for tumor cells that can survive without the proliferative stimulation from estrogen-ERα activation. One observed consequence of resistance is increased tumor aggressiveness [35]. Our studies suggest that similar mechanisms are occurring in the WPERRN mice, where ERα has been deleted, rather than pharmacologically antagonized. In short, this model genetically recapitulates some aspects of acquired chemotherapeutic resistance due to ER inhibition, namely less proliferative tumors with an increased metastatic capacity.

## Conclusions

In summary, the data herein demonstrate that Ron is overexpressed breast cancers with high levels or Ron observed in early stage ERα-positive breast cancers. Our studies also show that a conditional loss of ERα in mammary epithelium of Ron overexpressing mice leads to the development of breast tumors which exhibit diminished growth and increased metastases.

## Methods

### Animal Procedures

MMTV-Ron (FVB/N) mice were crossed to ERα^fl/fl ^(C57/B6) mice to generate ERα^fl/fl^/MMTV-Ron animals, abbreviated ERRN. Both transgenic strains are independently described elsewhere [13,18]. The ERRN mice were then crossed with transgenic mice carrying an allele for whey acidic protein promoter driving expression of Cre recombinase (WAP-Cre) [36,37] to create WAP-Cre/ERα^fl/fl^/MMTV-Ron animals, abbreviated WPERRN, plus littermate controls. Primers used for genotyping and recombination efficiency were described previously [13,18]. For tumor production and to ensure WAP-Cre expression, all females used in experiments were housed with males throughout the duration of the experiment to promote parity. All females were multiparous, and no differences in parity were observed between WPERRN and ERRN animals. Mice were palpated bi-weekly for determination of tumor latency. Tumors reaching 20% of body mass were considered end-stage, and animals were sacrificed. Athymic nude mice were purchased from NCI-Frederick (Frederick, MD). For orthotopic injections, 2 × 10^6 ^cells were suspended in a 1:1 mixture of DMEM:F12 and Matrigel^® ^(BD Biosciences) and injected into the fat pad of anesthetized mice. All procedures were approved by the University of Cincinnati Institutional Animal Care and Use Committee.

### Reagents

Primers used for genotyping mice were as follows: MMTV-Ron forward 5'TGG GTG GTG AGG TCT GCC AAC ATG A3', reverse 5'CCG TCT TCG GGA GTT AAA GAT CAG GG3'; ERα^fl/fl ^forward 5'TGG GTT GCC CGA TAA CAA TAA C3', reverse 5'AAG AGA TGT AGG GCG GGA AAA G3'; WAP-Cre forward 5'CAT CAC TCG TTG CAT CGA CC3', reverse 5'TAG AGC TGT GCC AGC CTC TTC3'. The ERα^fl/fl ^primers were also used for determining the deletion event in Cre-positive tumors and Cre-infected cells. Adenovirus expressing Cre and GFP or GFP described previously [22]. MTT reagent (Sigma, St. Louis, MO) was dissolved in PBS, added to the cells for 3 hours and the intensity of the resulting solution quantified on spectrophotometer at 570 nm.

### Immunoblotting and Immunohistochemistry

For immunoblotting, whole cell lysates were prepared in radioimmunoprecipitation assay (RIPA) buffer (20 mM Tris, 150 mM sodium chloride, 2 mM EDTA, 0.1% SDS, 1% Triton-X 100, 0.5% sodium deoxycholate, 10% glycerol) supplemented with Complete protease inhibitor tablets (Roche, Palo Alto, CA) and HALT phosphatase inhibitors (Pierce, Rockford, IL). Protein concentrations were determined by Micro BCA Protein Assays (Pierce, Rockford, IL). Lysates were then boiled for 10 minutes in buffer containing beta-mercaptoethanol and separated by SDS-PAGE. Immunoblotting was carried out according to standard procedures on PVDF membrane, with enhanced chemiluminescence detection (GE Healthcare, Piscataway, NJ). Antibodies used were the following: anti-mouse ERα (clone MC-20; Santa Cruz, Santa Cruz, CA), anti-human Ron β (clone C-20 Santa Cruz, Santa Cruz, CA), anti-mouse progesterone receptor (clone C-19, Santa Cruz), and anti-mouse cyclinD1 (clone 72-13G, Santa Cruz). Densitometry was performed on NIH ImageJ analysis software. For immunohistochemistry, tissues were fixed in neutral-buffered formalin, embedded in paraffin, and sectioned. Rehydrated sections were subjected to antigen retrieval and incubated in primary antibody overnight at 4°C. Sections were then, washed, incubated in biotin-conjugated goat secondary antibody for 1 hr at room temperature, and visualized with ABC-DAB kit (Vector Labs, Burlingame, CA).

### Quantitative real-time polymerase chain reaction analysis

Frozen tumor tissue from ERRN and WPERRN mice was homogenized and lysed in Tri-reagent (MRC, Cincinnati, OH). RNAs was isolated from Tri-reagent reagent solutions by chloroform-isopropanol extraction. DNA was prepared from isolated RNAs using High Capacity cDNA Reverse Transcriptase kit (Applied Biosystems, Foster City, CA), per manufacturer instructions. The sequences for primers are as follows: for CCND1 (5'CTC CTC TTC GCA CTT CTG CTC3', and 5'GCG TAC CCT GAC ACC AAT CTC3'), for SMAD3 (5' CCT TCT GGT GCT CCA TCT CC3', and 5'ACA CCT CTC CCA ATG TGT CG3'), for CTSD (5'CCT TTG ACA TCC ACT ACG GC3' and 5'AAG ATG CCA TCA AAC TTG GC3'). Expression of these genes was normalized to expression of β-glucuronidase (GUS, 5'TTG AGA ACT GGT ATA AGA CGC ATC AG3', 5'TCT GGT ACT CCT CAC TGA ACA TGC3'). Quantitative real-time PCR was performed using SYBR-green in 96-well plates read with 7900 HT Fast Real-Time PCR/Sequence Detection Systems (Applied Biosystems, Foster City, CA).

### Cell Based Assays

For migration assays, 1 × 10^5 ^ER^fl/fl^Ron or ER^-/-^Ron cells were plated in the top chamber of 0.8 μm transwells (Corning Costar Corporation, Cambridge, MA) and were allowed to migrate towards 5% FBS DMEM-F12 media for 24 hours. The number of live cells on the bottom of the transwell was measured using an MTT assay according to the manufacturer's instructions (Sigma-Aldrich, St. Louis, MO) with absorbance read at 570 nm. Invasion assays were performed similar to the migration assays except that the transwell inserts were coated with Matrigel™ (BD Biosciences, Billerica, MA) and invasion was assessed at 48 hrs. For cell death assays, cells were plated at equal densities followed by serum starvation for 48 hours. The cells, including the floating/dead cells, were collected and stained with AnnexinV and propidium iodide (BD Pharmingen, San Diego, CA) and analyzed by flow cytometry according to the manufacturer's instructions. Cell proliferation assays were accomplished by plating cells on glass cover slips. The cells were labeled for 4 hours with 5-bromo-2'-deoxyuridine (BrDU) and stained according to the manufacturer's instructions (RPN20, GE Healthcare Life Sciences, Piscataway, NJ). The number of BrDU positive cells and the total number of cells in a 10 × field were counted and represented as the percentage of BrDU positive cells per group.

### Tissue Microarray

The tissue arrays were purchased from the National Cancer Institute's Cooperative Breast Cancer Tissue Resource (CBCTR 2nd Generation Progression Tissue Microarray). Each array was purchased in duplicate, one serving as an isotype control for the immunohistochemistry that was performed as described above. Samples were scored as previously described for intensity on a 0-3 scale and for percent positivity (0-100%) [22,23]. The two parameters were multiplied to give an overall score (0-300). Blinded scoring was performed by two independent scorers, and the average score was calculated and used for each sample.

### Statistical Analysis

The statistical test for each experiment is listed in the corresponding figure legend. A p-value of < 0.05 is considered significant. All data were analyzed using Graphpad Prism software (LaJolla, CA).

## Competing interests

The authors declare that they have no competing interests.

## Authors' contributions

AMM and RJM were involved in the design, acquisition and analysis of the data as well as in drafting the manuscript. JKG, DG and KEL were involved in the acquisition of data. SAK was involved in the conception of the studies and the design of reagents for the ERα mouse model. SEW was involved in the conception and design of the study as well as in drafting and revising the manuscript. All authors have read and have approved the final manuscript.
